# Anti-SARS-CoV-2 IgG Antibody Response in Individuals Infected Post Complete Vaccination: A 6-Month Longitudinal Study in Healthcare Professionals

**DOI:** 10.3390/vaccines11061077

**Published:** 2023-06-08

**Authors:** Nicole Baratto, Lorenza Maistrello, Elena Pazienza, Rita Barresi

**Affiliations:** 1 Neurobiology Department, IRCCS San Camillo Hospital, Via Alberoni 70, 30126 Venice, Italy; nicolebaratto@yahoo.it (N.B.); elena.pazienza@hsancamillo.it (E.P.); 2 IRCCS San Camillo Hospital, Via Alberoni 70, 30126 Venice, Italy; lorenza.maistrello@hsancamillo.it

**Keywords:** antibody, COVID-19 vaccine, infection, healthy adults

## Abstract

Serological assays have been used to evaluate the magnitude of naturally acquired and BNT162b2 vaccine-induced immunity. In order to assess the extent to which the antibody response correlates with infection-mediated protection after vaccination, we investigated the kinetics of anti-SARS-CoV-2-S1 IgG in fully vaccinated healthy individuals who did or did not develop COVID-19 within 8 months after the booster dose. The anti-SARS-CoV-2-S1 receptor-binding, domain-specific IgG titer was assessed in serum samples collected at various intervals from 4 months after the second and 6 months after the third dose. The IgG level decreased 33% within 6 months after the second dose and, one month after the third dose, increased dramatically (>300%) compared with the pre-booster time point. COVID-19 infection within two months after the third dose did not cause significant IgG variation, but later viral infections elicited an IgG response similar to the initial response to the booster. The probability of developing COVID-19 and the severity of symptoms were not related to the antibody titer. Our data indicate that repeated exposure to viral antigens by either vaccination or infection at short-term intervals elicits limited boosting effects and that an IgG titer alone is not associated with the prediction of future infections and their symptomatology.

## 1. Introduction

COVID-19 is a disorder caused by Severe Acute Respiratory Syndrome Coronavirus 2 (SARS-CoV-2 virus). This novel coronavirus has a high transmissibility and spreads rapidly, and has resulted in a worldwide pandemic. To date, COVID-19 has affected over 700 million individuals and has been the cause of more than 6 million deaths worldwide. Infection with SARS-CoV-2 can cause a wide range of clinical manifestations, from no symptoms to multisystemic critical illness. Vaccination is an effective measure to reduce the severity of the acute disease and the occurrence of post-acute sequelae symptoms as well [1,2]. BNT162b2 (Pfizer–BioNTech) is one of the vaccines most administered worldwide. It is formulated as an RNA-lipid nanoparticle of nucleoside-modified mRNA encoding a P2 mutant spike protein that induces the production of spike-specific IgG [3]. Serological assays have been used to evaluate the magnitude of naturally acquired and BNT162b2-induced immunity. The antibody response within the vaccinated population has been monitored to identify low responders and priority groups for additional doses. It has been shown that humoral immunity significantly increases 4 to 6 weeks after the administration of the first dose of the vaccine and undergoes a remarkable decline approximately in 3 to 4 months after the second dose, with a 90% decrease of the antibody titer after 6 months [4,5,6]. Therefore, a third (booster) dose of the vaccine has been recommended to allow an increase of the immune response and to offer higher and longer-lasting protection against infection. Questions remain about the precise benefit of additional vaccination doses in the general population of healthy adults. Although there are numerous reports on immunoresponse in elderly and fragile individuals [7,8,9], only a few follow-up studies have been undertaken on younger (<60 year old) adults after the booster dose [10,11,12]. Importantly, infection may still occur after the full cycle of vaccinations, particularly with novel variants that develop a remarkable antibody evasion ability [13]. Further studies are also needed to assess the baseline of vaccine- and infection-elicited immunity response in healthy cohorts, particularly in order to identify patterns prognostic of Long-COVID-19 syndrome. This is a multisystem condition affecting many individuals, who continue to experience symptoms such as fatigue, neurocognitive difficulties, muscle pain, weakness, and depression for several months after the resolution of the acute disease and require multidisciplinary rehabilitative interventions, which has significant impact on public health [14,15].

In this study, we report the outcome of our single-center investigation of the kinetics of anti-SARS-CoV-2-S1 receptor-binding, domain (RBD)-specific antibodies (AB) up to 6 months after three doses of BNT162b2 in healthcare workers (HCW) both infection-naïve and infected after vaccination.

## 2. Materials and Methods

### 2.1. Study Cohort

Fifty-one HCW were recruited in this study (Table 1). All participants were generally healthy with no major comorbidities. An amount of 98% of the HCWs were Caucasian. Volunteers filled a form with information on age, gender, previous COVID-19 infections and symptomatology, date of vaccination, type, and duration of adverse effects. Two doses of the BNT162b2 vaccine were administered with a three-week dosing interval from January 2021. The booster dose was delivered between November and December 2021. All participants were screened for SARS-CoV-2 infection every 10–14 days by rapid antigen test, even in the absence of symptoms. Out of the 51 participants in this study, two had been infected and recuperated from SARS-CoV-2 infection before the first dose. No one was infected between the first and third dose. Twenty-six HCW were infected at various time points after the third dose. Among those, two contracted COVID-19 both before the first and after the third dose; three were infected twice after the third dose: one within one month and 6 months, one within 2 months and after 6 months, and the other within one month and 2 months. Most infections occurred between January and May 2022, when Omicron BA.4/5 variants were dominant in Europe. Genetic characterization of the variants was not performed.

### 2.2. Samples Collection and Analysis

Blood samples were collected in CAT serum tubes with a sep clot activator and allowed to clot at room temperature for at least 30 min. Subsequently, they were centrifuged for 15 min at 3000 rpm. The serum obtained was coded and aliquoted in small volumes (150–200 µL) and stored at −80 °C until use. Serum samples were obtained 4 (D2M4), 6 (D2M6), and pre-dose 3 (PRE), 9 months after the second dose, at which point the third dose was administered. Further samples were obtained 1 (D3M1), 2 (D3M2), 4 (D3M4), and 6 (D3M6) months after the booster. The AB concentration was assessed with ELISA SARS-CoV-2 anti-RBD IgG kit (EZRBDG-110K EMD Millipore Corporation, Billerica, MA, USA) according to the manufacturer’s instructions. The kit does not detect specific variants of SARS-CoV-2. The lower limit of quantitation of the assay is 1.56 ng/mL. A concentration value below 2.5 ng/mL was considered as a negative antibody titer and a value  >  2.5 ng/mL was considered as positive [16].

### 2.3. Statistical Analysis

Missing data for some of the variables (less than 25%) were imputed using the MICE (multivariate imputations by chain equations) method. Descriptive statistics (i.e., median, interquartile range, mean, standard deviation, and percentage) were used to summarize demographic and biological data. According to data distribution, evaluated by the Shapiro–Wilk test, a Spearman’s correlation test or a Pearson’s correlation test was performed to study the presence of associations between variables. A point-biserial correlation was used to investigate the correlation between antibody levels and qualitative variables (i.e., gender).

Differences in antibody levels at different time points were compared both in the general sample and by stratifying the sample according to whether or not they had contracted the virus (i.e., COVID-19 and non-COVID-19, respectively) using parametric (i.e., ANOVA for repeated measures) or non-parametric (i.e., Friedman) tests, depending on the distribution of data. In the case of significance parametric (i.e., Bonferroni test) or nonparametric (i.e., Wilcoxon test (V)), post hoc tests were performed. Significant differences between the samples after different doses at same time points (i.e., at 4 months after the second dose and 4 months after the third dose) were analyzed using parametric (e.g., paired-sample *t*-test) or nonparametric (e.g., Wilcoxon test) tests. The persistence of an AB response was investigated by tests for comparison (i.e., *t*-test or Mann–Whitney test).

AB levels between groups of subjects (COVID-19 and non-COVID-19) were compared for each time point by using the Kruskal–Wallis test (χ^2^). In the case of significance, nonparametric post hoc tests were performed (i.e., Mann–Whitney test (W)). Generalized regression models were used to assess the dynamics of AB (dependent variable) and relate these changes to previous infections (independent variables). For each regression model, goodness-of-fit was assessed by using the following indices: (i) McFadden’s index of explained variance (pseudo-R^2^); (ii) the Scaled Brier Score (sBS), which is a measure of overall accuracy and calculates the average prediction error; (iii) Construction of the ROC (Receiver Operating Characteristic) curve and evaluation of the Area Under the Curve (AUC); and (iv) the Hosmer–Lemeshow test for the fit between expected and estimated frequencies ($\chi_{\mathrm{HL}}^{2}$; P_HL_). The regression model fit the original data if the indices met the following criteria: (i) the more pseudo-R^2^ is next to 1, the more the model is satisfactory; (ii) Brier score for a model can range from 0 (0%) for a perfect model to 1 (100%) for a non-informative model; (iii) AUC values > 0.70 representing a moderately accurate model; (iv) a significant $\chi_{\mathrm{HL}}^{2}$ value indicating a bad model fit. All statistical analyses were performed using RStudio Version 2022.11.0 [17]. The statistical significance level was set to 0.05.

## 3. Results

**Figure 1:**
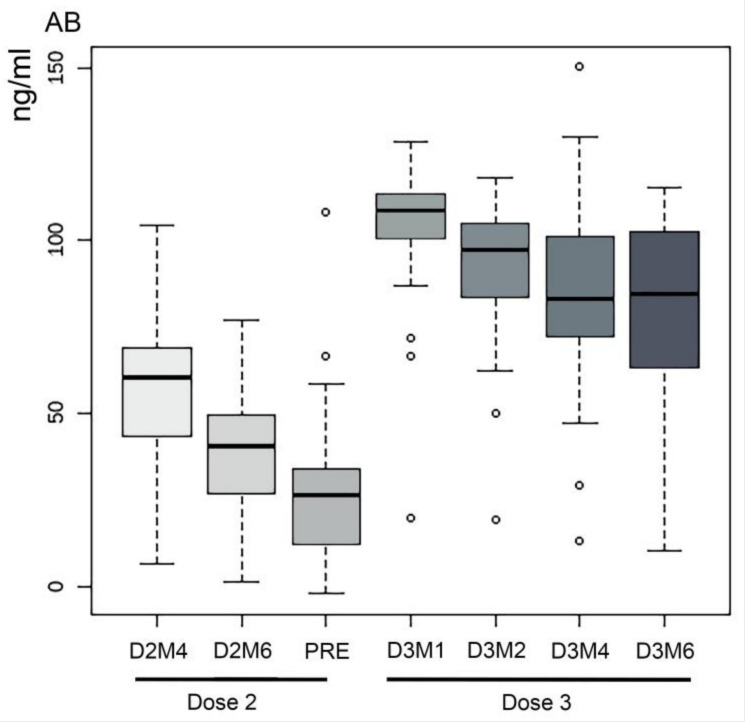
Boxplots of the distribution of the AB levels (ng/mL) of all participants at each time point.

Only two individuals showed AB levels below the cut-off for positive samples before PRE and all the samples were positive at all time points starting from D3M1. AB titer detected at D2M4, D2M6, and PRE in samples from two participants infected before the first dose was consistent with the average values of the cohort. Overall, a significant difference was found in the comparison of the AB titer (ng/mL) between D2M4 and D2M6 (V = 1323; *p* < 0.001), with decreasing AB levels (33%). In contrast, one month after the booster dose, the AB titer increased dramatically (>300%), compared with the pre-booster time point (PRE vs. D3M1; V = 1; *p* < 0.001). We observed an overall decline in AB concentration from D3M1 to D3M6: -11% between D3M1 and D3M2 (V = 1238; *p* < 0.001), -9% between D3M2 and D3M4 (V = 1132; *p* < 0.001), -6% between D3M4 and D3M6 (V = 903; *p* = 0.025) (Figure 1). No correlation was found between the variables Gender and Age and AB levels at different time points.

**Figure 2:**
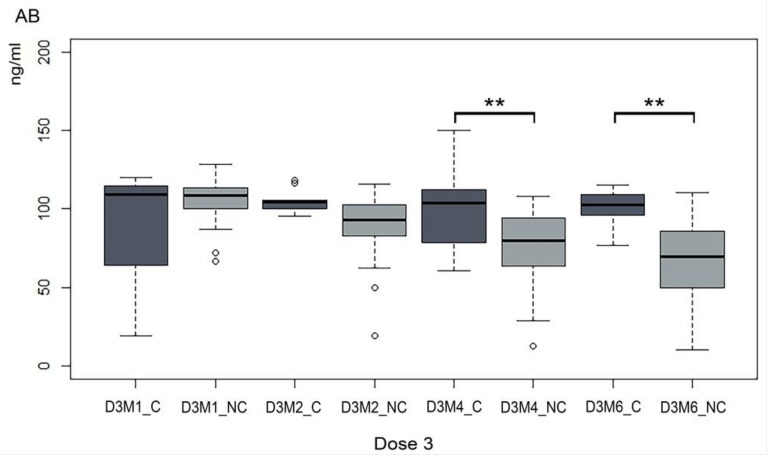
A comparative trend of the Ab titer in infected and not infected HCW. For each time point, antibody levels (ng/mL) were compared between those who had (C) and had not (NC) contracted SARS-CoV-2 up to that time point. D3M1_NC = no SARS-CoV-2 infection 1 months after booster; D3M1_C = SARS-CoV-2 infection 1 months after booster; D3M2_NC = no SARS-CoV-2 infection 2 months after booster; D3M2_C = SARS-CoV-2 infection 2 months after booster; D3M4_NC = no SARS-CoV-2 infection 4 months after booster; D3M4_C = SARS-CoV-2 infection 4 months after booster; D3M6_NC = no SARS-CoV-2 infection 6 months after booster; D3M6_C = SARS-CoV-2 infection 6 months after booster. Asterisks indicate significant differences ** = *p* < 0.05.

We then compared trends of the AB titer between not infected individuals (COVID-19 and non-COVID-19) and groups of individuals that had contracted SARS-CoV-2 after the booster dose (COVID-19: C) at each time interval, as indicated in Table 1. Differences in median AB levels between C and NC appear already from two months after the third dose (Figure 2). However, the differences were not statistically significant at early time points, showing that those who contracted SARS-CoV-2 before D3M2 maintained levels of AB similar to that detected at D3M1. Indeed, the Mann–Whitney test showed no significant differences in AB levels between the C and NC groups except at the time points prior to D3M4 and D3M6 (W = 561; *p* = 0.002 and W = 545; *p* < 0.001, respectively). A comparison of the initial AB response to the booster dose with AB following infection after D3M4 and D3M6 did not show substantial differences.

**Figure 3:**
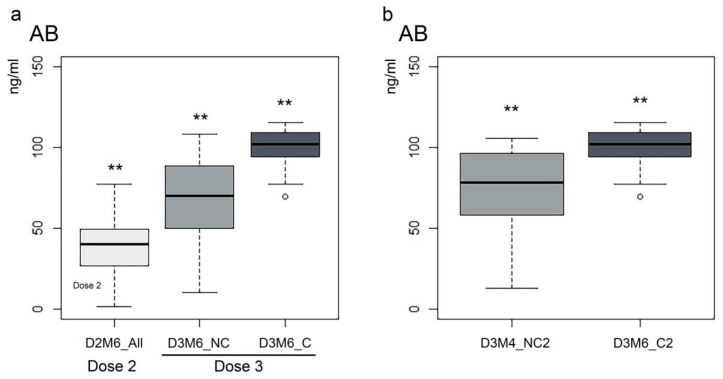
(**a**) The difference in AB levels (ng/mL) between all participants at 6 months after the second dose and the Non COVID-19 and COVID-19 groups at 6 months after the third dose. D2M6_All = all participants at 6 months after the second dose; D3M6_NC = participants who did not contract SARS-CoV-2 within 6 months after the third dose; D3M6_C = participants who contracted SARS-CoV-2 within 6 months after the booster dose. (**b**) The difference in AB levels between the NC2 group (subjects not infected) 4 months after the third dose and the C2 group (subjects who became infected within 2 months of the third dose) 4 months after the infection. Asterisks indicate significant differences ** = *p* < 0.05.

We then attempted to compare magnitude and persistence of the immunoresponse after the second and third vaccinations by comparing AB in all participants at D2M6 (All = 51) with participants who had never been infected (NC = 32) and those who had contracted SARS-CoV-2 during the study (C = 19). We observed that the median at D3M4 and D3M6 was higher (43% and 89%, respectively) than at the same time points after the second dose in the absence of COVID-19 infection (Figure 3a and not shown). Significant differences in AB levels between C and NC were found at D3M4 (W = 452.5; *p* = 0.006) and D3M6 (W = 538.5; *p* < 0.001), with higher AB levels (27% and 49%) in the C group (Figure 3a and not shown). Comparison of AB in all participants at D2M4 and D2M6 with C and NC D3M4 and D3M6 confirmed that the difference is significant among the three groups (respectively, χ^2^ = 32.33; *p* < 0.001 and χ^2^ = 52.84; *p* < 0.001), indicating that the immune response after 6 months remains more sustained after the third dose compared to two doses only, even if a breakthrough infection does not occur. In both cases, post hoc tests showed that all values were significantly different from each other with AB levels being higher in the C group.

We then inquired whether the viral infection in vaccinated individuals would generate a response comparable with a “4th dose” and therefore investigated the variation and persistence of the AB response following viral infection by comparing the AB titer 4 months after the booster dose to 4 months after infection. Eight individuals in our cohort were infected 2 months after the booster (group C2, 8/51); therefore, we compared AB levels measured in this group at D3M6 (4 months after infection) with AB levels measured in the non-infected cohort (group NC2, 43/51) at D3M4 (4 months after booster). As expected, we observed a significant difference (32%) with significantly higher values seen in the C2 group (W = 316; *p* = 0.0018) (Figure 3b). Three HCW in our cohort were infected twice within 6 months of receiving the booster. Analysis of individual AB kinetic showed a below-average response to the third dose only in one of them, who contracted SARS-CoV-2 after one month and then maintained an elevated AB titer similarly to other infected individuals.

**Figure 4:**
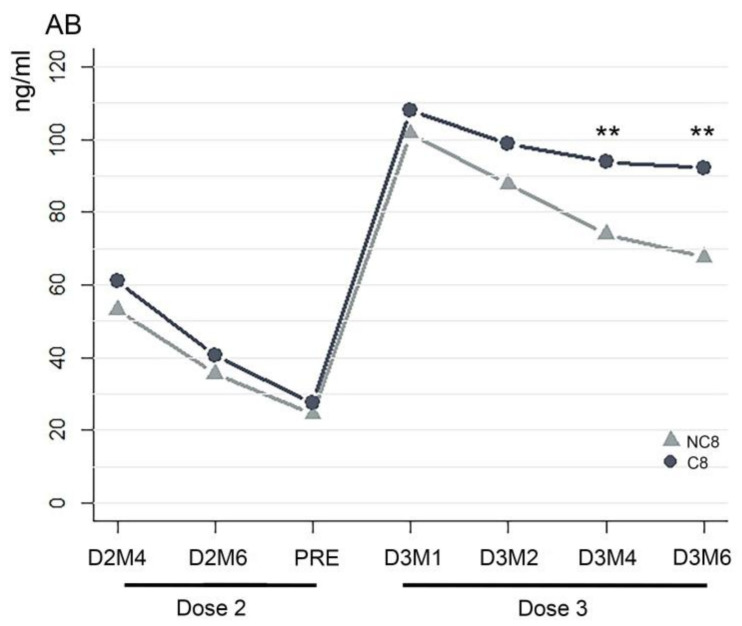
Comparison of AB titers between individuals infected and not infected after booster. The average AB titer (ng/mL) of subjects who became infected within 8 months after the third dose (COVID-19: C8) compared with that of the subjects who did not contract the virus (non-COVID-19: NC8) for all time points considered in the study. Asterisks indicate significant differences ** = *p* < 0.05.

Within two months after completion of the study (8 months after the third dose), seven more individuals reported to have contracted SARS-CoV-2. In order to test if the AB level can generally predict the susceptibility to infection, we included these into a larger COVID-19 infected cohort (C8, infected within 8 months after the booster; 26/51) and compared the average values of AB concentration between C8 and NC8, who remained not infected 8 months after the booster dose (25/51). Significant differences between groups were only seen at time points D3M4 (W = 477; *p* = 0.004) and D3M6 (W = 505.5; *p* < 0.001). Interestingly, the average values were slightly higher, although not significantly, in the infected C8 group at each interval before and after the booster (Figure 4).

To explore associations between AB level and probability of contracting the infection, we estimated a regression model with data obtained from 46/51 subjects using the SARS-CoV-2 infection as a response variable and values of AB levels corresponding to D2M4, D2M6, PRE, and D3M1 as regressors. The regression model showed that none of the selected variables was significantly associated with the dependent variable. However, the values of the indices used to assess the goodness of fit of the model, i.e., McFadden index (pseudo-R^2^), Scaled Brier Score (sBS), and Area Under the Curve (AUC) assessment, indicated that the data did not fit the estimated model well (pseudo-R^2^ = 0.005; AUC = 0.55; sBS = 0.005). Finally, we performed polyserial correlation analyses to investigate the presence of a correlation between the AB concentration and the severity of the clinical presentation after infection. The most reported symptoms were fatigue, cold, sore throat, cough, and fever. Mild sore throat, cold, and fatigue were classified as mild symptoms. Moderate symptoms were cough and mild cold and a fever that persisted up to 48 h. Severe symptoms were a more persistent high fever and cough. There was no correlation between the AB titer and severity of the symptoms (*p*-value > 0.05).

## 4. Discussion

Several vaccines for COVID-19 have been developed throughout the past years. The most widely available worldwide were made by using either messenger RNA (mRNA) (Pfizer–BioNTech, Moderna) or adenoviral vectors (Janssen–Johnson & Johnson, Oxford/AstraZeneca, Sputnik-V). An inactivated whole-virus SARS-CoV-2 vaccine (e.g., Sinovac, Sinopharm) and a type containing a synthetic version of the S protein (Novavax) were also generated but comparatively less commonly used in western countries [18]. Reports investigating the immunogenicity of different types of vaccines are overall in agreement, demonstrating their efficacy. Direct inter-laboratory comparison can, however, rarely be made due to a lack of standardized anti-SARS-CoV-2 quantitative and neutralizing assays [19]. Comparative studies in cohorts of recipients of two doses of different vaccines showed that Pfizer/BioNTech and Sputnik V vaccines induce a greater antibody response and persistence than Sinopharm and Sinovac vaccines [20,21]. Reports on AB response after three doses of vaccine mainly monitor fragile populations undergoing specific therapies [9,22] or follow up healthy adult cohorts for a relatively short time [23,24,25]. With rare exceptions [26], they focus on cohorts inoculated by means of BNT162b2 or heterologous vaccination protocols [27,28]. The latter studies are of particular interest, since in spring 2021, it was recommended that individuals that had received a vector vaccine should complete vaccinations with an mRNA vaccine. Assessment of the antibody response generated by homologous BNT162b2 vaccination with that generated by various heterologous vaccination regimens showed equivalent results [29]. Interestingly, a study comparing the antibody response to BNT162b2, Moderna, Oxford/AstraZeneca, and Janssen–Johnson & Johnson following primary and booster vaccinations identified the lowest spike-specific IgG levels after one dose of Janssen–Johnson & Johnson, intermediate levels after two doses of BNT162b2, and the highest levels after two doses of the Moderna vaccine. These differences, however, were evened out after the vaccination with a booster dose [30]. To our knowledge, reports of kinetics of the immune response in heterologous regimens that encompass more than a few weeks after the booster dose are still scarce.

Our study of the humoral immune response of RBD-spike IgG AB after completion of SARS-CoV-2 vaccination series in the period ranging from 4 months after the second dose to 6 months after the third dose confirmed that the booster induces an AB response more sustained over time than the second dose [10,11,25,31,32,33]. Furthermore, in the course of the study, 26 volunteers (51%) contracted SARS-Cov-2 at various time points after the administration of the booster. Not surprisingly, the average of AB levels in the convalescent group were higher than the concentration in the naïve group. Notably, a statistically significant difference was observed in the months following the period in which the greatest number of infections occurred (4 and 6 months after the third dose, when the Omicron BA.4/5 variant was prevalent). In addition, our data not only confirm that the AB titer is not correlated to age and sex but also demonstrate that the probability of being infected and the severity of symptoms are not related to age, sex, and the AB titer of the participants. Moreover, the time since vaccination was not a strong determinant against infection. Our data analysis did not take an account of other covariants (e.g., BMI, lifestyle or type of work, comorbidities such as diabetes, cancer, etc.) that were the objects of studies by other groups [28,34,35]. Those were in agreement, demonstrating a reduced antibody response in older individuals or in patients with comorbidities or high or low BMI. In relatively homogeneous groups of healthy HCW, like the cohort in our study, age and BMI did not seem to be affecting the strength of the immune response significantly [28].

Remarkably, we observed that the AB titer following viral infection within 2 months and after 4 and 6 months remained or returned substantially similar to the initial response to the booster dose, although the waning in the AB titer appeared delayed in the infected individuals up to 4 months after acute exposure to the viral antigen compared to the non-infected cohort. These findings are analogous to other studies carried out in healthy subjects, where it was highlighted that: (i) AB levels of infected and non-infected subjects were not significantly different, (ii) AB concentration increases with the vaccine booster and/or infection, but can reach a plateau [10,31]. Likewise, other reports have shown that the immune system stimulation by the administration of a booster dose shortly after the natural infection or the last vaccine dose can lead to a limited increase in the humoral response (ceiling of immunity) [36]. Therefore, if we may compare the infection to an additional dose of vaccine administered a few weeks after the third dose, our observations indicate that repeated exposure to viral antigens either by vaccination or by infection at short-term intervals elicits a limited boosting effect. This suggests that three doses may be sufficient to achieve the maximal immunogenic effect of the vaccine in healthy individuals.

Although we were not able to identify any correlations between the AB concentration and the possibility of acquiring the infection, it must be highlighted that the continuous evolution of SARS-CoV-2 determines structural changes that allow the emerging variants to partially evade the immune response generated by the vaccine or by previous infections. Our methodology did not allow detection of AB to specific variants; however, studies have shown that the booster stimulates IgG affinity, with a positive impact in neutralization capacity [33]. The secretion of high-avidity antibodies will likely induce higher protection against novel variants. Wei et al. [37] have demonstrated that the booster dose increased the protection against Omicron infection but, at the same antibody titer, previous Delta/Omicron BA.1 infection provided higher protection against Omicron BA.4/5 compared with previous Pre-Alpha/Alpha infection. Importantly, a previous Omicron BA.2 infection gave the highest protection against Omicron BA.4/5 infection. Indeed, it has been shown that vaccinated individuals infected with the BA.1 variant developed strong neutralizing antibodies against the BA.1 and the BA.2 variants, but fewer against BA.2.12.1 and BA.4/BA.5. Remarkably, it appears that the neutralizing activity against Omicron BA.4/5 in BA.1 convalescent individuals relied mostly on RBD binding antibodies, while N-terminal domain binding antibodies were driving the neutralizing activity in BA.2 convalescent individuals [38]. Overall, it is anticipated that the infection with other variants may confer a higher protection against novel infections than just three doses of the vaccine [39]. As new variants will continue to develop, determining how infection with different sub variants might protect against the new ones is critical for the development of adapted vaccines.

An intriguing retrospective observation is that before and immediately after receiving the third vaccination, the median AB response in subjects who eventually became infected was slightly higher in comparison with those who remained COVID-19 negative up to 8 months after the booster. This trend is interesting but not statistically significant and indicates that the AB concentration alone is not correlated with the prediction of possible future infections. In fact, only two out of the three participants who became infected multiple times during the study presented an AB concentration slightly lower than the average of the other participants at the time interval prior to the first infection. Surprisingly, too, is that two other individuals in our cohort that presented with a very low AB titer never became infected, despite reporting having been at close and prolonged contact with infected individuals. Since the immune system is a complex machine, the combined analysis of a panel of heterogeneous biomarkers should give better predictive insight and provide a more detailed picture of the immunological context of healthy subjects in response to vaccine and infection. Indeed, it is now clear that serum AB, although an indicator in serological tests, is not an effective marker in predicting the response of the immune system following a full cycle of vaccination against SARS-CoV-2 [40]. The contact of the antigen with the components of the immune system, in fact, determines the activation of both humoral and cell-mediated immunity that play key roles in the individual response, which is also influenced by genetic factors [40]. In addition, extensive biomarker studies might help to shed light on predisposing and pathogenic features of Long-COVID syndrome, which seems to be linked to hyper inflammation that leads to immune dysregulation. Features of cytokine storm syndrome have been reported [41], which cause multisystemic deleterious effects, such as neuropsychiatric symptoms [42]. Although the incidence of Long-COVID as a consequence of infection with Omicron seems to be lower than after infection with the Delta variants [43], numerous occurrences of post-COVID-19 symptoms are still documented. Efforts are focused on assessing the prevalence and classifying the clinical outcomes by clinical signs [44]. As neurocognitive and psychiatric sequelae are among the most frequent manifestations of post-COVID-19 syndromes, neurorehabilitation interventions have become major targets of studies and clinical trials [45,46]. Further investigations are required in order to evaluate the significance of individual occurrences, diagnose emergent conditions, and tailor a preventive rehabilitative plan.

Our study has certain limitations: (i) the modest number of participants, (ii) only a wild-type IgG (S-RBD) evaluation with one commercial test that does not allow the determination of levels of antibodies in international units (BAU/mL), (iii) lack of functional data on AB or assays measuring neutralizing IgG. These limitations may be addressed in further studies. On the other hand, this study also has the strengths of a relatively extended follow up of AB monitoring of a controlled population regularly screened for SARS-CoV-2 infection and the opportunity to compare infection-naïve and infected individuals in a relatively homogeneous cohort.

## 5. Conclusions

Overall, our findings confirm the effective immunogenic action of the third dose of the BNT162b2 vaccine and verify that repeated exposure to viral antigens by either vaccination or infection at short-term intervals elicits a limited boosting effect on the AB titer. Furthermore, the AB titer alone is not a predictive biomarker of future infections and their symptomatology.

## Figures and Tables

**Table 1:** Participants’ demographics and SARS-CoV-2 infection events after third dose.

Sample (N = 51)	
Sex, males/females, n (%)	16 (31%)/35 (69%)
Age, years, mean ± SD	36.47 ± 9.45
SARS-CoV-2 status, Positive/Negative, n (%)	26 (51%)/25 (49%)
SARS-CoV-2 infection events after 3rd dose (N = 26 + 3 *)	
Within 2 months, n (%)	9/29(31%)
Within 2–4 months, n (%)	3/29 (10%)
Within 4–6 months, n (%)	9/29 (31%)
After 6 months, n (%)	8/29 (28%)

* Three volunteers contracted the infection twice after the third dose.

## Data Availability

Not applicable.

## References

[1] Al-Aly Z., Agarwal A., Alwan N., Luyckx V.A. (2023). Long COVID: Long-term health outcomes and implications for policy and research. Nat. Rev. Nephrol..

[2] Azzolini E., Levi R., Sarti R., Pozzi C., Mollura M., Mantovani A., Rescigno M. (2022). Association between BNT162b2 Vaccination and Long COVID after Infections Not Requiring Hospitalization in Health Care Workers. JAMA.

[3] Walsh E.E., Frenck R.W., Falsey A.R., Kitchin N., Absalon J., Gurtman A., Lockhart S., Neuzil K., Mulligan M.J., Bailey R. (2020). Safety and Immunogenicity of Two RNA-Based COVID-19 Vaccine Candidates. N. Engl. J. Med..

[4] Padoan A., Cosma C., Bonfante F., Della Rocca F., Barbaro F., Santarossa C., Dall’Olmo L., Pagliari M., Bortolami A., Cattelan A. (2022). Neutralizing antibody titers six months after Comirnaty vaccination: Kinetics and comparison with SARS-CoV-2 immunoassays. Clin. Chem. Lab. Med..

[5] Favresse J., Bayart J.L., Mullier F., Elsen M., Eucher C., Van Eeckhoudt S., Roy T., Wieers G., Laurent C., Dogné J.M. (2021). Antibody titres decline 3-month post-vaccination with BNT162b2. Emerg. Microbes Infect..

[6] Jo D.H., Minn D., Lim J., Lee K.D., Kang Y.M., Choe K.W., Kim K.N. (2021). Rapidly Declining SARS-CoV-2 Antibody Titers within 4 Months after BNT162b2 Vaccination. Vaccines.

[7] Gilboa M., Mandelboim M., Indenbaum V., Lustig Y., Cohen C., Rahav G., Asraf K., Amit S., Jaber H., Nemet I. (2022). Early Immunogenicity and Safety of the Third Dose of BNT162b2 Messenger RNA Coronavirus Disease 2019 Vaccine Among Adults Older Than 60 Years: Real-World Experience. J. Infect. Dis..

[8] Bensouna I., Caudwell V., Kubab S., Acquaviva S., Pardon A., Vittoz N., Bozman D.F., Hanafi L., Faucon A.L., Housset P. (2022). SARS-CoV-2 Antibody Response after a Third Dose of the BNT162b2 Vaccine in Patients Receiving Maintenance Hemodialysis or Peritoneal Dialysis. Am. J. Kidney Dis..

[9] Shroff R.T., Chalasani P., Wei R., Pennington D., Quirk G., Schoenle M.V., Peyton K.L., Uhrlaub J.L., Ripperger T.J., Jergović M. (2021). Immune responses to two and three doses of the BNT162b2 mRNA vaccine in adults with solid tumors. Nat. Med..

[10] Kontopoulou K., Nakas C.T., Papazisis G. (2022). Significant Increase in Antibody Titers after the 3rd Booster Dose of the Pfizer-BioNTech mRNA COVID-19 Vaccine in Healthcare Workers in Greece. Vaccines.

[11] Rastawicki W., Juszczyk G., Gierczyński R., Zasada A.A. (2022). Comparison of anti-SARS-CoV-2 IgG and IgA antibody responses post complete vaccination, 7 months later and after 3rd dose of the BNT162b2 vaccine in healthy adults. J. Clin. Virol..

[12] Uwamino Y., Yokoyama T., Sato Y., Shibata A., Kurafuji T., Tanabe A., Noguchi M., Arai T., Ohno A., Yokota H. (2023). Humoral and cellular immune response dynamics in Japanese healthcare workers up to six months after receiving a third dose of BNT162b2 monovalent vaccine. Vaccine.

[13] Xiang T., Wang J., Zheng X. (2022). The humoral and cellular immune evasion of SARS-CoV-2 Omicron and sub-lineages. Virol. Sin..

[14] De Luca R., Bonanno M., Calabrò R.S. (2022). Psychological and Cognitive Effects of Long COVID: A Narrative Review Focusing on the Assessment and Rehabilitative Approach. J. Clin. Med..

[15] Nalbandian A., Sehgal K., Gupta A., Madhavan M.V., McGroder C., Stevens J.S., Cook J.R., Nordvig A.S., Shalev D., Sehrawat T.S. (2021). Post-acute COVID-19 syndrome. Nat. Med..

[16] Sukumaran A., Thomas R.E., Krishnan R.A., Thomas T., Thomas R., Vijayan D.K., Paul J.K., Vasudevan D.M. (2022). Sequential Profiling of Anti-SARS CoV-2 IgG Antibody in Post COVID-19 Patients. Indian. J. Clin. Biochem..

[17] Team R.C. (2022). R: A Language and Environment for Statistical Computing.

[18] Graña C., Ghosn L., Evrenoglou T., Jarde A., Minozzi S., Bergman H., Buckley B.S., Probyn K., Villanueva G., Henschke N. (2022). Efficacy and safety of COVID-19 vaccines. Cochrane Database Syst. Rev..

[19] Infantino M., Pieri M., Nuccetelli M., Grossi V., Lari B., Tomassetti F., Calugi G., Pancani S., Benucci M., Casprini P. (2021). The WHO International Standard for COVID-19 serological tests: Towards harmonization of anti-spike assays. Int. Immunopharmacol..

[20] Dashdorj N.J., Wirz O.F., Röltgen K., Haraguchi E., Buzzanco A.S., Sibai M., Wang H., Miller J.A., Solis D., Sahoo M.K. (2021). Direct comparison of antibody responses to four SARS-CoV-2 vaccines in Mongolia. Cell Host Microbe.

[21] Ebrahim F., Tabal S., Lamami Y., Alhudiri I.M., El Meshri S.E., Al Dwigen S., Arfa R., Alboeshi A., Alemam H.A., Abuhtna F. (2022). Anti-SARS-CoV-2 IgG Antibodies Post-COVID-19 or Post-Vaccination in Libyan Population: Comparison of Four Vaccines. Vaccines.

[22] Giambra V., Piazzolla A.V., Cocomazzi G., Squillante M.M., De Santis E., Totti B., Cavorsi C., Giuliani F., Serra N., Mangia A. (2022). Effectiveness of Booster Dose of Anti SARS-CoV-2 BNT162b2 in Cirrhosis: Longitudinal Evaluation of Humoral and Cellular Response. Vaccines.

[23] Panico A., Lobreglio G., Bagordo F., Zizza A., De Donno A., Rosato C., Lazzari R., Chicone M., Indino F., Recchia V. (2022). Antibody Response in Healthcare Workers before and after the Third Dose of Anti-SARS-CoV-2 Vaccine: A Pilot Study. Vaccines.

[24] Salerno G., Gentile G., De Luca O., Costanzi G., Cirelli G., Di Simone Di Giuseppe B., Marcellini L., Anibaldi P., Marcolongo A., Simmaco M. (2022). Age-Related Dynamics of Serum Anti-Spike IgG Ab after the Third Dose of BNT162b2 Vaccine in a Naive Health Care Workers Population. Viral Immunol..

[25] Kwon S.R., Kim N., Park H., Minn D., Park S., Roh E.Y., Yoon J.H., Shin S. (2022). Strong SARS-CoV-2 Antibody Response after Booster Dose of BNT162b2 mRNA Vaccines in Uninfected Healthcare Workers. J. Korean Med. Sci..

[26] Zeng G., He F., Zhang X., Li G., Wang X., Gan Y., Zheng C., Tang J., Xu L., Zhao J. (2023). Antibody response to the third dose of inactivated COVID-19 vaccine in people living with HIV (PLWH): A longitudinal cohort. J. Med. Virol..

[27] Wanlapakorn N., Kanokudom S., Phowatthanasathian H., Chansaenroj J., Suntronwong N., Assawakosri S., Yorsaeng R., Nilyanimit P., Vichaiwattana P., Klinfueng S. (2023). Comparison of the reactogenicity and immunogenicity between two-dose mRNA COVID-19 vaccine and inactivated COVID-19 vaccine followed by an mRNA vaccine in children aged 5–11 years. J. Med. Virol..

[28] Montero S., Urrunaga-Pastor D., Soto-Becerra P., Cvetkovic-Vega A., Guillermo-Roman M., Figueroa-Montes L., Sagástegui A.A., Alvizuri-Pastor S., Contreras-Macazana R.M., Apolaya-Segura M. (2023). Humoral response after a BNT162b2 heterologous third dose of COVID-19 vaccine following two doses of BBIBP-CorV among healthcare personnel in Peru. Vaccine X.

[29] Rose R., Neumann F., Grobe O., Lorentz T., Fickenscher H., Krumbholz A. (2022). Humoral immune response after different SARS-CoV-2 vaccination regimens. BMC Med..

[30] Hvidt A.K., Baerends E.A.M., Søgaard O.S., Stærke N.B., Raben D., Reekie J., Nielsen H., Johansen I.S., Wiese L., Benfield T.L. (2022). Comparison of vaccine-induced antibody neutralization against SARS-CoV-2 variants of concern following primary and booster doses of COVID-19 vaccines. Front. Med..

[31] Ogrič M., Žigon P., Podovšovnik E., Lakota K., Sodin-Semrl S., Rotar Ž., Čučnik S. (2022). Differences in SARS-CoV-2-Specific Antibody Responses after the First, Second, and Third Doses of BNT162b2 in Naïve and Previously Infected Individuals: A 1-Year Observational Study in Healthcare Professionals. Front. Immunol..

[32] Terpos E., Karalis V., Sklirou A.D., Apostolakou F., Ntanasis-Stathopoulos I., Bagratuni T., Iconomidou V.A., Malandrakis P., Korompoki E., Papassotiriou I. (2022). Third dose of the BNT162b2 vaccine results in very high levels of neutralizing antibodies against SARS-CoV-2: Results of a prospective study in 150 health professionals in Greece. Am. J. Hematol..

[33] Lustig Y., Gonen T., Meltzer L., Gilboa M., Indenbaum V., Cohen C., Amit S., Jaber H., Doolman R., Asraf K. (2022). Superior immunogenicity and effectiveness of the third compared to the second BNT162b2 vaccine dose. Nat. Immunol..

[34] Piernas C., Patone M., Astbury N.M., Gao M., Sheikh A., Khunti K., Shankar-Hari M., Dixon S., Coupland C., Aveyard P. (2022). Associations of BMI with COVID-19 vaccine uptake, vaccine effectiveness, and risk of severe COVID-19 outcomes after vaccination in England: A population-based cohort study. Lancet Diabetes Endocrinol..

[35] Zhao M., Slotkin R., Sheth A.H., Pischel L., Kyriakides T.C., Emu B., McNamara C., Shi Q., Delgobbo J., Xu J. (2022). Clinical Variables Correlate with Serum Neutralizing Antibody Titers after COVID-19 mRNA Vaccination in an Adult, US-based Population. medRxiv.

[36] Noh J.Y., Cheong H.J., Kim W.J., Choi J.Y., Lee H.W., Kim S.S., Kim B., Song J.Y. (2022). Robust neutralizing antibody responses after single-dose BNT162b2 vaccination at long intervals from prior SARS-CoV-2 infection and ceiling effect with repeated vaccination. J. Infect..

[37] Wei J., Matthews P.C., Stoesser N., Newton J.N., Diamond I., Studley R., Taylor N., Bell J.I., Farrar J., Kolenchery J. (2023). Protection against SARS-CoV-2 Omicron BA.4/5 variant following booster vaccination or breakthrough infection in the UK. Nat. Commun..

[38] Muik A., Lui B.G., Bacher M., Wallisch A.K., Toker A., Finlayson A., Krüger K., Ozhelvaci O., Grikscheit K., Hoehl S. (2022). Omicron BA.2 breakthrough infection enhances cross-neutralization of BA.2.12.1 and BA.4/BA.5. Sci. Immunol..

[39] Yamamoto S., Mizoue T., Ohmagari N. (2023). Analysis of Previous Infection, Vaccinations, and Anti-SARS-CoV-2 Antibody Titers and Protection against Infection with the SARS-CoV-2 Omicron BA.5 Variant. JAMA Netw. Open.

[40] Cheetham N.J., Kibble M., Wong A., Silverwood R.J., Knuppel A., Williams D.M., Hamilton O.K.L., Lee P.H., Bridger Staatz C., Di Gessa G. (2023). Antibody levels following vaccination against SARS-CoV-2: Associations with post-vaccination infection and risk factors in two UK longitudinal studies. Elife.

[41] Cron R.Q., Caricchio R., Chatham W.W. (2021). Calming the cytokine storm in COVID-19. Nat. Med..

[42] Mazza M.G., De Lorenzo R., Conte C., Poletti S., Vai B., Bollettini I., Melloni E.M.T., Furlan R., Ciceri F., Rovere-Querini P. (2020). Anxiety and depression in COVID-19 survivors: Role of inflammatory and clinical predictors. Brain Behav. Immun..

[43] Antonelli M., Pujol J.C., Spector T.D., Ourselin S., Steves C.J. (2022). Risk of long COVID associated with delta versus omicron variants of SARS-CoV-2. Lancet.

[44] Mizrahi B., Sudry T., Flaks-Manov N., Yehezkelli Y., Kalkstein N., Akiva P., Ekka-Zohar A., Ben David S.S., Lerner U., Bivas-Benita M. (2023). Long COVID outcomes at one year after mild SARS-CoV-2 infection: Nationwide cohort study. BMJ.

[45] Rolin S., Chakales A., Verduzco-Gutierrez M. (2022). Rehabilitation Strategies for Cognitive and Neuropsychiatric Manifestations of COVID-19. Curr. Phys. Med. Rehabil. Rep..

[46] Hagen B.I., Lerdal A., Søraas A., Landrø N.I., Bø R., Småstuen M.C., Becker J., Stubberud J. (2022). Cognitive rehabilitation in post-COVID-19 condition: A study protocol for a randomized controlled trial. Contemp. Clin. Trials.

